# Silicea

**Published:** 1898-01

**Authors:** 


### HOMŒOPATHIC PHYSICIAN,

A MONTHLY JOURNAL OF

HOMŒOPATHIC MATERIA MEDICA AND CLINICAL MEDICINE.

'If our school ever gives up the strict inductive method of Hahnemann, we are lost, and deserve only to be mentioned as a caricature in the history of medicine."—CONSTANTINE HERING.

Vol. XVIII. JANUARY, 1898. No. 1.

### EDITORIAL.

SILICEA.—No remedy in the materia medica is more surprising and more clearly shows the genius of Hahnemann than Silicea.

That the "everlasting rocks" can be used as medicines seems incredible and absurd. Silicea has been a stumbling-block with many to the acceptance of the homœopathic doctrine. It has been argued that if rock can withstand the weather for centuries, it is incredible that it can be used as a medicine. It must be inert, and therefore cannot influence the human system. This argument is known to have been used by a professor before a class in a homœopathic college. Of course, it is not true. The weather does disintegrate the rocks, and in comparatively few years makes very considerable changes in them. Silicea or Silica is known to be feebly soluble in water. Fresenius, the great German analytical chemist, has declared that water standing for an hour or two in a tumbler will dissolve off a perceptible amount of Silica.

The method of preparing Silica as a medicine is not by simple trituration of sand in a mortar with sugar of milk, as was once stated by a homœopathic professor. Whoever will take the trouble to read Hahnemann's *Chronic Diseases* will see that the directions given by Hahnemann for its preparation insure its solubility. These directions are to fuse pure quartz or rock crystal with soda, in a crucible. This makes a glass which is soluble in water, and is hence called water-glass. The solution is made, and then the soda is removed by muriatic acid, when the silica falls down as a thick, heavy jelly. Water has taken the place of the soda, and the compound is no longer a silicate of soda but a silicate of water, or, as it is usually named, "Hydrated Silica" or "Hydrated Silicic-acid."

This hydrated silica is then easily made up into a homœopathic remedy, either by trituration or dilution. Silica occurring in the soil or in rocks is constantly being broken down and dissolved by the alkalies, potash, soda and ammonia, and so rendered accessible to plants, by which they are enabled to build it up into their own structure. A conspicuous example of this utilization by plants of the silica in the soil for their own structure is the stalk that supports an ear of wheat. The greater part of its composition is silica, which gives to the slender tube its stiffness and capacity to bear the weight of so many grains of wheat.

In Greenland is found a white mineral called cryolite. It is a fluoride of aluminium and sodium. It is therefore rich in soda. Vast quantities of this mineral are brought to Philadelphia, where the soda is combined with silica to make a water-glass. This water-glass is thick and brownish yellow, and has the consistency of treacle, and looks like it. It is sold in great quantities for washing wool and for other purposes where soap would be ordinarily used.

The silica in the water-glass holds pretty much the same relation to the alkali that the fatty acids in soap do. It moderates the intensity of action of the alkali, so that this water-glass may be called a mineral soap.

These statements are made to remove the prejudice existing in many minds against the use of Silica in medicine on account of its supposed inertness.

Those who have used Silicea in practice, and have been made familiar by experience with its magnificent clinical qualities, will not need the foregoing explanations, and will not be any more influenced by them than before reading this editorial. There are, however, others who have not this fortunate clinical knowledge, but whose minds are filled instead with the erroneous teaching of men who are more than half hostile to the homœopathic principle they claim to practice as well as teach. To them these statements are particularly addressed.

